# Dual-Emission Fluorescence Resonance Energy Transfer (FRET) PCR Discriminates *Salmonella* Pullorum and Gallinarum

**DOI:** 10.3390/microorganisms12091815

**Published:** 2024-09-02

**Authors:** Jiansen Gong, Nneka Vivian Iduu, Di Zhang, Kelly Chenoweth, Lanjing Wei, Yi Yang, Xinhong Dou, Chengming Wang

**Affiliations:** 1 Key Laboratory for Poultry Genetics and Breeding of Jiangsu Province, Jiangsu Institute of Poultry Sciences, Yangzhou 225125, China; jjsensen@163.com (J.G.); shawn_zd@126.com (D.Z.); 2 Department of Pathobiology, College of Veterinary Medicine, Auburn University, Auburn, AL 36849, USA; nvi0001@auburn.edu (N.V.I.);; 3 Bioengineering Program, The University of Kansas, Lawrence, KS 66045, USA; lanjingwei@ku.edu; 4 College of Veterinary Medicine, Yangzhou University, Yangzhou 225009, China; yangyi@yzu.edu.cn; 5 Jiangsu Co-Innovation Center for the Prevention and Control of Important Animal Infectious Disease and Zoonose, Yangzhou University, Yangzhou 225009, China; douxinhong611@163.com

**Keywords:** *Salmonella* Pullorum, *Salmonella* Gallinarum, FRET-PCR, SNP, *pegB* gene, differentiation

## Abstract

*Salmonella* Pullorum (*S.* Pullorum) and *Salmonella* Gallinarum (*S.* Gallinarum) are two biovars of *Salmonella enterica* serovar Gallinarum, responsible for pullorum disease and fowl typhoid, which are the most prevalent and pathogenic forms of salmonellosis in poultry in developing countries. Traditional differentiation methods for *S*. Pullorum and *S*. Gallinarum are based on distinct clinical manifestations and biochemical traits, given their indistinguishable nature via serological assays alone. Molecular differentiation methods such as allele-specific PCR and dual PCR combined with gel electrophoresis or enzyme digestion have also been used to discriminate *S*. Pullorum and *S*. Gallinarum, but the detection efficiency is not high. This investigation introduces a Fluorescence Resonance Energy Transfer (FRET) PCR assay targeting the *pegB* gene, exclusively found in specific *Salmonella* serovars such as *S*. Pullorum and *S*. Gallinarum, and exhibiting conserved single-nucleotide polymorphisms across these two biovars. High-resolution melting curve analysis demonstrates distinct dissolution profiles, facilitating the precise discrimination of *S*. Pullorum and *S*. Gallinarum. This FRET-PCR assay exhibits a detection limit of 10 copies per reaction and has been rigorously validated utilizing 17 reference strains and 39 clinical isolates. The innovation presented herein provides a valuable tool for the rapid differentiation of *S*. Pullorum and *S*. Gallinarum, thereby enhancing diagnostic efficiency and molecular surveillance of poultry *Salmonella*. The developed *pegB*-targeting FRET-PCR assay presents a promising alternative to current cumbersome and time-consuming diagnostic modalities, offering significant potential for expedited identification and control of *Salmonella* in poultry and mitigating economic losses associated with *Salmonella* contamination in poultry production.

## 1. Introduction

*Salmonella* is the most common zoonosis pathogen in Enterobacteriaceae and *Salmonella* food poisoning represents a significant proportion of bacterial foodborne illnesses, posing considerable public health concerns. Globally, the number of food poisoning cases attributed to *Salmonella* infection remains alarmingly high, with non-typhoidal *Salmonella* infections causing approximately 681,316 deaths and 3.4 million illnesses [1]. A 2010 World Health Organization survey report on foodborne diseases worldwide highlights that *Salmonella* ranks first among 22 bacterial, viral, and protozoan diseases [2].

Poultry is recognized as the primary reservoir for *Salmonella enterica*, and human infections are often linked to the consumption of contaminated poultry meat and eggs [3]. Multiple *Salmonella enterica* serovars can infect poultry, with *Salmonella enterica* serovar Gallinarum being the most prevalent and pathogenic serovar, especially in developing countries [4]. *S*. Gallinarum exhibits host specificity, exclusively infecting chickens and turkeys, leading to severe systemic diseases and substantial economic losses [5]. Based on disease characteristics, *S*. Gallinarum can be further classified into two biovars, Pullorum and Gallinarum, causing pullorum disease and fowl typhoid in poultry, respectively [6]. Given the significant impact of these two pathogens on poultry production, they are crucial to poultry salmonellosis, requiring global elimination efforts.

Traditional serological typing methods face challenges differentiating between these two biovars due to their antigenic similarities. Additionally, biochemical identification techniques are limited in their usefulness, as they do not meet the needs for rapid and large-scale detection. However, with advancements in molecular biology, nucleic acid-based detection techniques for *Salmonella* have been developed, offering significantly faster detection times and improved efficiency compared to traditional methods.

Previous research has identified a highly conserved Peg fimbriae operon in the genomes of *S*. Pullorum and *S*. Gallinarum. This operon primarily comprises four structural genes, *pegA*, *pegB*, *pegC*, and *pegD*, encoding fimbriae major subunits, chaperones, ushers, and fimbriae adhesins, respectively [7]. This study aimed to establish a FRET-PCR method for rapidly identifying and differentiating these two biovars.

## 2. Material and Methods

### 2.1. Reference Bacterial Strains

This study included a total of 17 reference strains: ten *S*. Pullorum (ATCC19945, ATCC10398, CMCC50771, CVCC519, CVCC521, CVCC526, CVCC527, CVCC529, CVCC530, CVCC533) and seven *S*. Gallinarum (ATCC9184, CVCC79301, CVCC536, CVCC537, CVCC538, CVCC539, 9R). The reference strains used in this work were obtained from the American Type Culture Collection (ATCC), National Center for Medical Culture Collections (CMCC), and China Veterinary Culture Collection Center (CVCC), respectively.

### 2.2. Genomic DNA Extraction

Bacterial genomic DNA from bacterial culture was extracted using the Bacterial Genomic DNA Extraction Kit (TIANGEN, Beijing, China) following the manufacturer’s instructions. The extracted DNA was eluted in 100 μL of Tris-EDTA (TE) buffer [10 mmol L^−1^ Tris, 0.1 mmol L^−1^ EDTA (pH 8.0)] and stored at −80 °C until further analysis.

### 2.3. Differential FRET-PCR Targeting PegB Gene

#### Primers and Probes

The *pegB* gene is exclusively present in certain *Salmonella* serovars [7] and is universally found in *S*. Pullorum and *S*. Gallinarum. BLASTn using the *pegB* nucleotide sequences from *S*. Pullorum (CP012347) showed no significant similarity when excluding the organism *Salmonella*. The *pegB* nucleotide sequences of representative *S*. Pullorum (CP012347, LK931482, CP075018, CP012347, CP006575) and *S*. Gallinarum (CP019035, CP118112, CP118116, CP088142, CP077760) were obtained from GenBank. The Clustal Multiple Alignment Algorithm analysis using Vector NTI 11.0 (North Bethesda, MD, USA) demonstrated that there are, in total, three single nucleotide polymorphisms (SNPs) in the *pegB* gene across these two biovars, while each SNP is highly conserved among each strain of *S*. Pullorum and *S*. Gallinarum. At nucleotide positions 54, 70, and 581 of the *peg* gene, *S*. Pullorum has the nucleotides T (Thymine), G (Guanine), and A (Adenine), respectively, while *S*. Gallinarum has the nucleotides C (Cytosine), A (Adenine), and C (Cytosine). Based on this, a 100% match region was designed for the upstream and downstream primers, while the relatively conserved region with two SNPs was used as the probe region, enabling the differentiation of *S*. Pullorum and *S*. Gallinarum through high-resolution melting curve analysis. The FRET-PCR was designed to cover the *peg* SNP positions 54 and 70.

The primers and probes were synthesized by Integrated DNA Technologies (Coralville, IA, USA) [8]. The differential *Salmonella* FRET-PCR using Vector NTI 11.0 (North Bethesda, MD, USA) amplifies a 131 bp target with the following primers and probes: forward primer: 5′-TGGATGATTGCATTATGCCT-3′; downstream primer: 5′-CGTTTACCGTCATTCATTAA-3′; anchor probe: 5′-TGCCTGCGTGGAGCGGCATTT-6-FAM-3′; reporter probe: 5′-cy5-TATATATGGTACACGTATTATTTATCCGG-Phosphate-3′. The fluorescein probe was 3′-labeled with carboxyfluorescein (6-FAM), which acts as the FRET donor probe, excited by 488 nm light. The cy5 probe was HPLC-purified and used as the FRET acceptor probe, emitting ∼640 nm fluorescence following excitation by 6-FAM in close physical proximity.

Thermal cycling differential *Salmonella* FRET-PCR was performed in a LightCycler^®^ 480 II real-time PCR platform (Roche Diagnostics, Indianapolis, IN, USA). Each reaction was performed in a 20 μL final volume containing 10 μL of extracted DNA, as described by Wang et al. and DeGraves et al. [9,10]. Thermal cycling consisted of 18 high-stringency step-down cycles followed by 30 relaxed-stringency fluorescence acquisition cycles. The 18 high-stringency step-down thermal cycles were 6 × 10 s at 95 °C, 12 s at 64 °C, 8 s at 72 °C; 9 × 10 s at 95 °C, 12 s at 62 °C, 8 s at 72 °C; 3 × 10 s at 95 °C, 12 s at 60 °C, 8 s at 72 °C. The relaxed-stringency fluorescence acquisition cycling consisted of 30 × 10 s at 95 °C, followed by fluorescence acquisition of 12 s at 56 °C and 10 s at 72 °C. Once the FRET-PCR was completed, the melting curve analysis for probes annealing to the PCR products was determined by monitoring the fluorescence from 45 °C to 80 °C, and the first derivatives of F4/F1 were evaluated to determine the probe melting temperature (*T*_m_). Both DNA strands of the PCR products were sequenced at the Genomic Sequencing Laboratory (Sangon, Shanghai, China) using the forward and downstream primers.

### 2.4. Testing of PegB-Targeting FRET-PCR with Reference Strains and Clinical Isolates

Plasmids used for analytical sensitivity (LOD): Three plasmids (Integrated DNA Technologies, Coralville, IA, USA) containing portions of the *pegB* gene of *S*. Pullorum and *S*. Gallinarum were used as the positive controls and for quantitative standards (10^5^, 10^4^, 10^3^, 10^2^, 10^1^ copies of *pegB* molecules/10 μL).

Calculating coefficients of variability (CVs): FRET-qPCR on three replicates of quantitative standards (10^5^, 10^4^, 10^3^, 10^2^, 10^1^ copies per reaction) of *S.* Gallinarum and *S.* Pullorum was performed to calculate the CV%. The CV% for each standard = [standard deviation (SD) of the means/mean of plate means] × 100. The overall % CV is the average % CV for each of these five standards.

Analytical specificity based on reference strains: The specificity of the FRET-PCR assay was established by testing the Genomic DNA of 17 *Salmonella* reference strains. In addition, the PCR products were verified with DNA sequencing (Sangon, Shanghai, China).

Clinical isolates: A total of 739 unexposed dead chicken embryos (from 11 chicken farms in eight regions of Jiangsu Province, China) collected from March to December 2023 were used in this study. The yolk sac or viscera of the dead embryos were added to Selenite Brilliant Green (SBG) broth (HopeBio, Qingdao, China) under sterile conditions and incubated overnight at 37 °C. The enriched liquid was coated with Xylose Lysine Desoxycyclate (XLD) agar (HopeBio, Qingdao, China) and incubated at 37 °C for 24 h. The isolates identified as *Salmonella enterica* serovar Gallinarum were selected for dulcitol fermentation and ornithine decarboxylation tests to differentiate between the biovars Pullorum and Gallinarum. Simultaneously, purified bacteria were grown overnight at 37 °C in 5 mL of Luria–Bertani (LB) broth, then 1 mL of culture medium was used for genomic DNA extraction, followed by validation using our FRET-PCR assay.

DNA Sequencing Both strands of DNA of the PCR products were directly sequenced on an ABI 3730 DNA sequencer at the Genomic Sequencing Laboratory (Sangon, Shanghai, China) using the forward and downstream primers.

## 3. Results and Discussion

**Figure 1:**
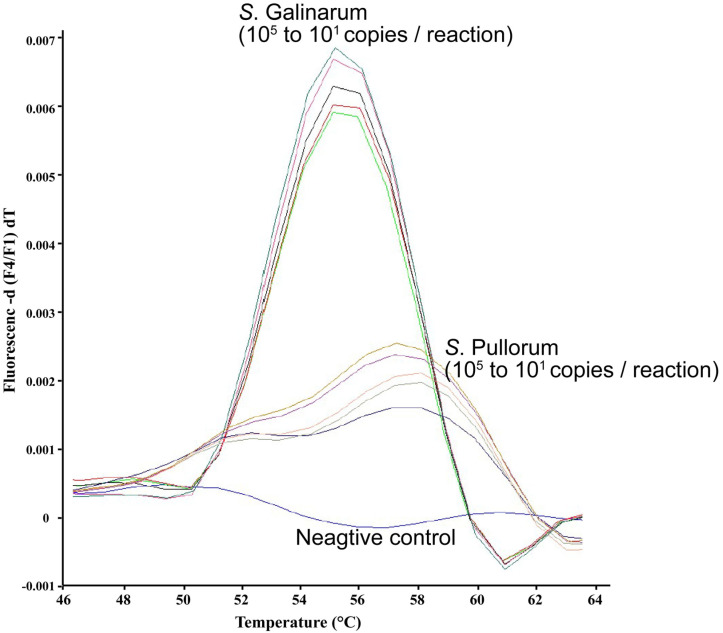
Discrimination of *S.* Pullorum and *S.* Gallinarum through high-resolution melting curve analysis genotyping. The *T*_m_ of probe hybridization to the targets was determined by high-resolution melting (HRM) curve analysis as the peak of the second derivative of the fluorescence released during a temperature increase from 38 °C to 85 °C. Fluorescence demonstrates unique and distinct *T*_m_ differences between *S.* Pullorum (59.6 °C with wide shoulder ± standard deviation 0.17 °C), *S.* Gallinarum (56.2 °C ± standard deviation 0.16 °C), and negative control (no melting curve). When five concentrations of the targets (10^1^ to 10^5^ copies of gene/reaction system, illustrated in different lines) were used for each *Salmonella* strain, the peaks and shapes of the melting curves remained the same.

Our *pegB*-targeting FRET-PCR demonstrated high sensitivity, detecting as few as 10 genomic copies of *S*. Pullorum and *S*. Gallinarum (Figure 1). The correlation coefficient (r) was −0.99 and −0.96 for *S*. Gallinarum and *S*. Pullorum, respectively. The CV% was found to be 0.83 (SD: 0.76) for *S*. Gallinarum and 1.23 for *S*. Pullorum (SD: 0.43). The % CV in this system is well below 10, the acceptable value [11]. Nucleotide mismatches between the probes and the PCR amplicons generated from *S*. Pullorum and *S*. Gallinarum led to unique and distinguishable melting curve analyses in the PCR, showing distinct *T*_m_ values for these two biovars (Figure 1). Specifically, S. Gallinarum exhibited a lower melting temperature of 56.2 °C with a sharp shoulder, while *S*. Pullorum exhibited a higher melting temperature of 59.6 °C with a wide shoulder. Furthermore, the peaks and shapes of the melting curves remained consistent across five concentrations of the targets (10^1^ and 10^5^ copies of gene per reaction system) used in this FRET-PCR.

To further validate the performance of our FRET-PCR methods, 17 reference strains and 39 clinical isolates were analyzed using the FRET-PCR assay and traditional bacteriological identification methods. For the reference strains, all of the *S*. Pullorum and *S*. Gallinarum displayed specific amplification curves and facilitated identification. As presented in Table 1, 39 strains of *Salmonella enterica* serovar Gallinarum (37 *S*. Pullorum and 2 *S*. Gallinarum) were identified. The positive rates for *S*. Pullorum and *S*. Gallinarum were 5.0% (37/739) and 0.3% (2/739), respectively. The results obtained from the FRET-PCR assay were in complete agreement with those obtained using traditional bacteriological identification methods (Table 1). The most important *Salmonella* serovar in poultry worldwide is *Salmonella enterica* serovar Gallinarum, which poses significant economic losses in poultry production. Although *Salmonella enterica* serovar Gallinarum is well-controlled in commercial flocks in developed countries, it remains a significant concern in developing countries [12].

Rapid, sensitive, and easily performed tests to detect and differentiate *S.* Pullorum and *S.* Gallinarum are essential for controlling the rapid spread of these pathogens. Current methods for this purpose are generally cumbersome and time-consuming. For instance, differentiating between *S.* Pullorum and *S.* Gallinarum often requires sophisticated methods such as allele-specific PCR based on the *rfbS* gene [13], dual PCR or multiplex real-time PCR based on the *speC* and *glgC* genes [14,15], PCR based on the variable region of the *ratA* gene [16], and PCR-RFLP assays based on the *fliC* or *fimH* genes [17,18]. Moreover, these methods often necessitate follow-up experiments such as gel electrophoresis or enzyme digestion.

Addressing this diagnostic challenge, our study established a FRET-PCR targeting the *pegB* gene, which is highly conserved in *S.* Pullorum and *S.* Gallinarum. Notably, the highly conserved *pegB* gene exhibited three unique SNPs. The probes in our study were designed based on regions containing two of these SNPs, resulting in distinct melting temperatures. Validation using reference strains and clinical isolates, followed by DNA sequencing, confirmed that the established FRET-PCR can differentiate these two important biovars in poultry with high sensitivity and can conveniently differentiate them within a single PCR system, eliminating the need for gel electrophoresis or DNA sequencing.

The accuracy and reliability of the FRET-PCR assay were validated against traditional bacteriological identification methods using a comprehensive set of reference strains and clinical samples. The results from the FRET-PCR assay were in complete agreement with those obtained from traditional methods, highlighting its potential as a rapid and efficient diagnostic tool.

FRET-PCR, or Fluorescence Resonance Energy Transfer PCR, utilizes dual-labeled probes that emit fluorescence upon hybridization to the target sequence. This technique offers enhanced specificity by detecting fluorescence only when the probe binds to the target, making it ideal for genotyping and SNP analysis [19,20].

The FRET-PCR used in this study offers the advantage of higher specificity due to the use of dual-labeled probes, which reduces the likelihood of non-specific amplification. This method is particularly useful for genotyping and SNP detection. However, FRET PCR requires designing and optimizing specific probes for each target, making it more labor-intensive and costly compared to SYBR Green PCR [20].

On the other hand, SYBR Green PCR is a cost-effective and straightforward method that relies on the intercalating dye SYBR Green to detect amplified DNA in real time. It allows for a wide range of applications, including gene expression analysis and mutation detection. However, SYBR Green PCR is more prone to non-specific amplification and may require additional steps, such as melting curve analysis, to confirm the specificity of the amplified product [20].

In conclusion, our developed *pegB*-targeting FRET-PCR assay offers a promising alternative to current cumbersome and time-consuming diagnostic methods. This method could significantly contribute to the rapid identification and control of *Salmonella* in poultry and reduce economic losses associated with *Salmonella* contamination in poultry production.

## Figures and Tables

**Table 1:** Detection of *S.* Pullorum *and S.* Gallinarum in clinical isolates.

Farm	Region	No. of Samples	*PegB* FRET-PCR Identification		Bacteriological Identification	
			*S*. Pullorum	*S*. Gallinarum	*S*. Pullorum	*S*. Gallinarum
A	Jiangdu	307	21		21	
B	Gaoyou	91	4		4	
C	Baoying	59	6		6	
D	Peixian	58		2		2
E	Yizheng	57	1		1	
F	Wujin	45	4		4	
G	Lishui	41				
H	Haian	32				
I	Peixian	22				
J	Jiangdu	16				
K	Peixian	11	1		1	
Total		739	37	2	37	2

## Data Availability

Data are contained within the article.
